# Phytosphingosine Alleviates Cigarette Smoke‐Induced Bronchial Epithelial Cell Senescence in Chronic Obstructive Pulmonary Disease by Targeting the Free Fatty Acid Receptor 4

**DOI:** 10.1002/mco2.70345

**Published:** 2025-08-29

**Authors:** Yuan Zhan, Zhesong Deng, Ruonan Yang, Shanshan Chen, Jiaheng Zhang, Yating Zhang, Hao Fu, Qian Huang, Yiya Gu, Zhilin Zeng, Jinkun Chen, Jixian Zhang, Jixing Wu, Jungang Xie

**Affiliations:** ^1^ Department of Respiratory and Critical Care Medicine National Clinical Research Center of Respiratory Disease, Key Laboratory of Pulmonary Diseases of Health Ministry, Tongji Hospital, Tongji Medical College, Huazhong University of Science and Technology Wuhan Hubei China; ^2^ Department of Respiratory and Critical Care Medicine The First Affiliated Hospital of Chongqing Medical University Chongqing China; ^3^ Department and Institute of Infectious Diseases Tongji Hospital, Tongji Medical College and State Key Laboratory for Diagnosis and Treatment of Severe Zoonotic Infectious Diseases, Huazhong University of Science and Technology Wuhan China; ^4^ Lawrence Bloomberg Faculty of Nursing University of Toronto Toronto Ontario Canada; ^5^ Department of Respiratory and Critical Care Medicine Hubei Provincial Hospital of Integrated Chinese & Western Medicine Wuhan Hubei China

**Keywords:** cellular senescence, chronic obstructive pulmonary disease, cigarette smoke, free fatty acid receptor 4, phytosphingosine

## Abstract

Chronic obstructive pulmonary disease (COPD) is a complex and irreversible respiratory disorder with a poor prognosis and a lack of effective pharmaceutical treatment. Our previous metabolomics study identified phytosphingosine (PHS) as a key differential metabolite in COPD that is positively correlated with lung function. In this study, we investigated the bioactive effects of PHS on experimental COPD and its underlying mechanisms using cigarette smoke (CS)‐induced mouse and cell models. We found that administering PHS improved CS‐induced lung dysfunction, emphysema, and airway inflammation by reducing cellular senescence and the senescence‐associated secretory phenotype in bronchial epithelium. Mechanistically, PHS interacted with the free fatty acid receptor 4 (FFAR4) and upregulated its expression, leading to the modulation of STIP1 homology and U‐Box containing protein 1 (STUB1) downstream, which controlled the ubiquitination levels of P53 and mitigated cellular senescence. Moreover, both FFAR4 overexpression through intratracheal injection of adeno‐associated virus and the administration of the FFAR4 agonist TUG891 showed therapeutic effects on CS‐induced lung damage. Our results highlight the beneficial impacts of PHS in experimental COPD mediated through the FFAR4 receptor, protecting against CS‐induced bronchial epithelial cell senescence and suggesting PHS as a promising therapeutic agent for COPD.

## 1 Introduction

Chronic obstructive pulmonary disease (COPD) is a progressive inflammatory lung disorder with significant morbidity and mortality, imposing a substantial global socioeconomic burden [1]. Aside from COVID‐19‐related deaths, COPD ranks as the third leading cause of mortality, affecting over 3 million individuals annually, with limited effective treatments available [2]. Clinically, COPD manifests with persistent respiratory symptoms and irreversible airflow limitation, characterized by worsening airway inflammation and emphysema [3]. The onset of COPD is influenced by intricate interactions between genetic and environmental factors, with chronic cigarette smoke (CS) inhalation serving as the primary risk factor [1, 4]. Recent evidence indicates a link between COPD and accelerated lung aging, evidenced by the accumulation of senescent cells and elevated levels of the senescence‐associated secretory phenotype (SASP), which significantly impact disease progression, severity, and prognosis in COPD patients [5, 6]. Nonetheless, the precise mechanisms underlying lung aging in COPD remain incompletely understood.

The bronchial epithelium is crucial for defense against harmful particles, with structural or functional abnormalities implicated in COPD pathogenesis [7]. Cellular senescence, a hallmark of aging, involves the irreversible arrest of the cell cycle due to stressors, which hinders tissue repair, compromises physiological integrity, causes organ dysfunction, and alters secretion patterns [8]. Recent research has highlighted the significant role of cellular senescence in bronchial epithelial cells in the inflammatory response associated with COPD [9]. Chronic CS exposure can induce oxidative DNA damage, triggering cellular senescence via the P53/P21/P16 pathway. Senescent cells remain metabolically active and secrete proinflammatory mediators, known as SASP, including interleukins, chemokines, and matrix metalloproteinases, thereby driving the progression of COPD [5, 10]. Therefore, elucidating the molecular mechanisms of cellular senescence in COPD and investigating potential anti‐aging interventions may mitigate the disease burden.

Metabolic disturbance is a prominent feature of COPD, closely linked to inflammation and the immune system. Studies have shown elevated levels of betaine and choline in urine, as well as an increased ratio of carnitine to acylcarnitine in the serum of COPD patients compared to healthy individuals [11, 12]. Analysis of induced sputum from COPD participants revealed decreased levels of free alpha‐linolenic acid, linoleic acid, and eicosapentaenoic acid [13]. Our previous study has identified differences in glycerophospholipid and amino acid metabolism between COPD patients and controls based on metabolomic analyses of lung tissue and plasma samples [14]. Additionally, higher levels of phosphatidylcholine 34:3 were positively correlated with COPD progression and decline in lung function, specifically forced expiratory volume in 1 s (FEV1) [15]. These findings collectively confirm the presence of metabolic abnormalities in COPD, particularly in lipid metabolism, and suggest potential roles of specific metabolites in mitigating COPD severity.

In our previous study [14], we identified phytosphingosine (PHS) as a differentially expressed phospholipid molecule in the lung tissues of patients with COPD. PHS is predominantly expressed in epithelial tissues such as the epidermis and gastrointestinal tract [16], and is known for its various physiological functions, including anti‐inflammatory effects (e.g., suppression of proinflammatory factors like NO and TNF‐α), antitumor activity (inducing apoptosis and G2/M phase arrest in lung cancer cells), and metabolic regulation (activation of PPARα to enhance glucose and lipid metabolism) [16, 17, 18]. Through these mechanisms, PHS has been implicated in the pathogenesis of inflammatory and neoplastic diseases [17, 19, 20], but its role in COPD and CS‐related cellular senescence remains unclear. Therefore, this current study aims to investigate the role and underlying molecular mechanisms of PHS in mitigating CS‐induced bronchial epithelial cellular senescence, improving histopathological damage, and enhancing lung function in COPD. This research may offer a novel therapeutic approach for managing COPD in clinical settings.

## 2 Results

### 2.1 Phytosphingosine Improved Lung Dysfunction, Emphysema, Airway Inflammation, and Senescence of Bronchial Epithelium in CS‐Exposed Mice

**FIGURE 1:**
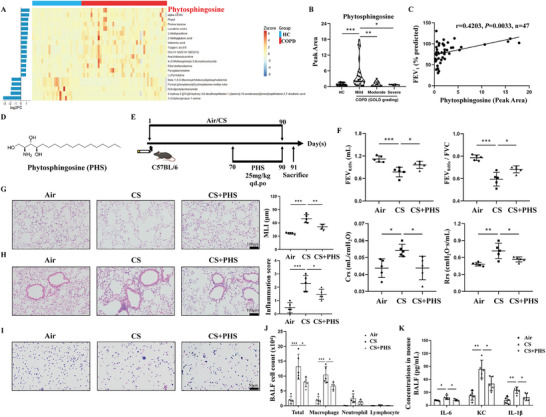
Phytosphingosine improved lung dysfunction, emphysema, airway inflammation in CS‐exposed mice. **(A)** Heat map of top 20 differential metabolites from the metabolomics result of human lung tissues, including 47 COPD patients and 27 controls. **(B)** PHS levels of lung tissues among different GOLD‐grading groups. **(C)** Correlation analysis of PHS and lung function in COPD patients. **(D)** The chemical structure of phytosphingosine. **(E)** CS‐induced COPD‐model mice and the method of PHS delivery. **(F)** The results of lung function including FEV_0.05s_, FEV_0.05S_/FVC, Crs, and Rrs. **(G)** The representative images of HE‐stained lung sections across the alveolar area and the semi‐quantitative results of MLI indicating the emphysema extent. Scale bar = 100 µm, magnification = 200x. **(H)** The representative images of HE‐stained lung sections around the airway and the results of inflammation score. Scale bar = 100 µm, magnification = 200x. **(I)** The representative images of Liu's staining against various cells in mouse BALF. **(J)** The numbers of total cells, macrophages, neutrophils, and lymphocytes in mouse BALF. **(K)** ELISA results of IL‐6, KC, and IL‐1β levels in BALF supernatant. Data were expressed as mean ± SD. *p*‐values were calculated using one‐way ANOVA. *p < 0.05, **p < 0.01, and ***p < 0.001 represented significant differences. BALF, bronchoalveolar lavage fluid; COPD, chronic obstructive pulmonary disease; Crs, compliance of respiratory system; CS, cigarette smoke; FEV1, forced expiratory volume in 1 s; FEV_0.05s_, forced expiratory volume in 0.05 s; FVC, forced vital capacity; GOLD, Global Initiative for Objective Assessment of Lung Disease; HC, healthy control; HE, hematoxylin‐eosin; MLI, mean linear intercept; PHS, phytosphingosine; Rrs, respiratory system resistance.

In our previous study [14], PHS was identified as a novel metabolic biomarker for distinguishing between individuals with COPD and healthy controls using non‐targeted metabolomic analysis. Subsequent analysis of lung tissue metabolomic data revealed that PHS levels were significantly elevated in COPD patients compared to healthy controls (Figure 1A). Furthermore, when classifying COPD patients into three groups based on the Global Initiative for Chronic Obstructive Pulmonary Disease (GOLD) classification, we observed that PHS levels peaked in the mild group and decreased as disease severity increased (Figure 1B). Additionally, PHS showed a positive correlation with lung function in COPD patients (Figure 1C), suggesting a potential protective role of PHS in the progression of COPD.

To investigate the role of PHS in COPD, we established a CS‐exposure mouse model with or without the oral administration of PHS (its chemical structure is shown in Figure 1D; the simplified diagram of drug administration in Figure 1E). We first evaluated the potential toxic effects of PHS by assessing liver, kidney, and cardiac function markers, finding no significant differences in alanine aminotransferase (ALT), aspartate aminotransferase (AST), urea nitrogen (BUN), creatinine (CR), lactate dehydrogenase (LDH), and creatine kinase (CK) among the groups. Additional histopathological examinations by H&E staining also revealed no observable pathological changes, such as cellular degeneration, inflammatory infiltration, or structural disruption, in any of these organs (Figure S1). Lung function tests revealed significantly lower forced expiratory volume in 0.05 s (FEV_0.05s_) and FEV_0.05_/forced vital capacity (FVC), and higher compliance of respiratory system (Crs) and respiratory system resistance (Rrs) in the CS group compared to the Air group. However, CS‐exposed mice treated with PHS showed improvements in these parameters (Figure 1F). Subsequent histopathological analysis indicated reduced lung destruction in the CS+PHS group compared to the CS group, as evidenced by decreased mean linear intercept (MLI) values (Figure 1G), suggesting a potential protective effect of PHS against CS‐induced emphysema.

In addition, we also investigated the impact of PHS on airway inflammation. Mice in the CS+PHS group showed reduced infiltration of inflammatory cells around the airways and lower inflammation scores compared to the CS group (Figure 1H). The PHS‐treated mice exhibited significantly decreased total and various inflammatory cell counts, particularly macrophages, in bronchoalveolar lavage fluid (BALF) compared to the CS group (Figures 1I,J). Furthermore, ELISA analysis of BALF supernatant revealed significantly lower levels of IL‐6, KC, and IL‐1β in CS‐exposed mice receiving PHS compared to those exposed only to CS (Figure 1K).

**FIGURE 2:**
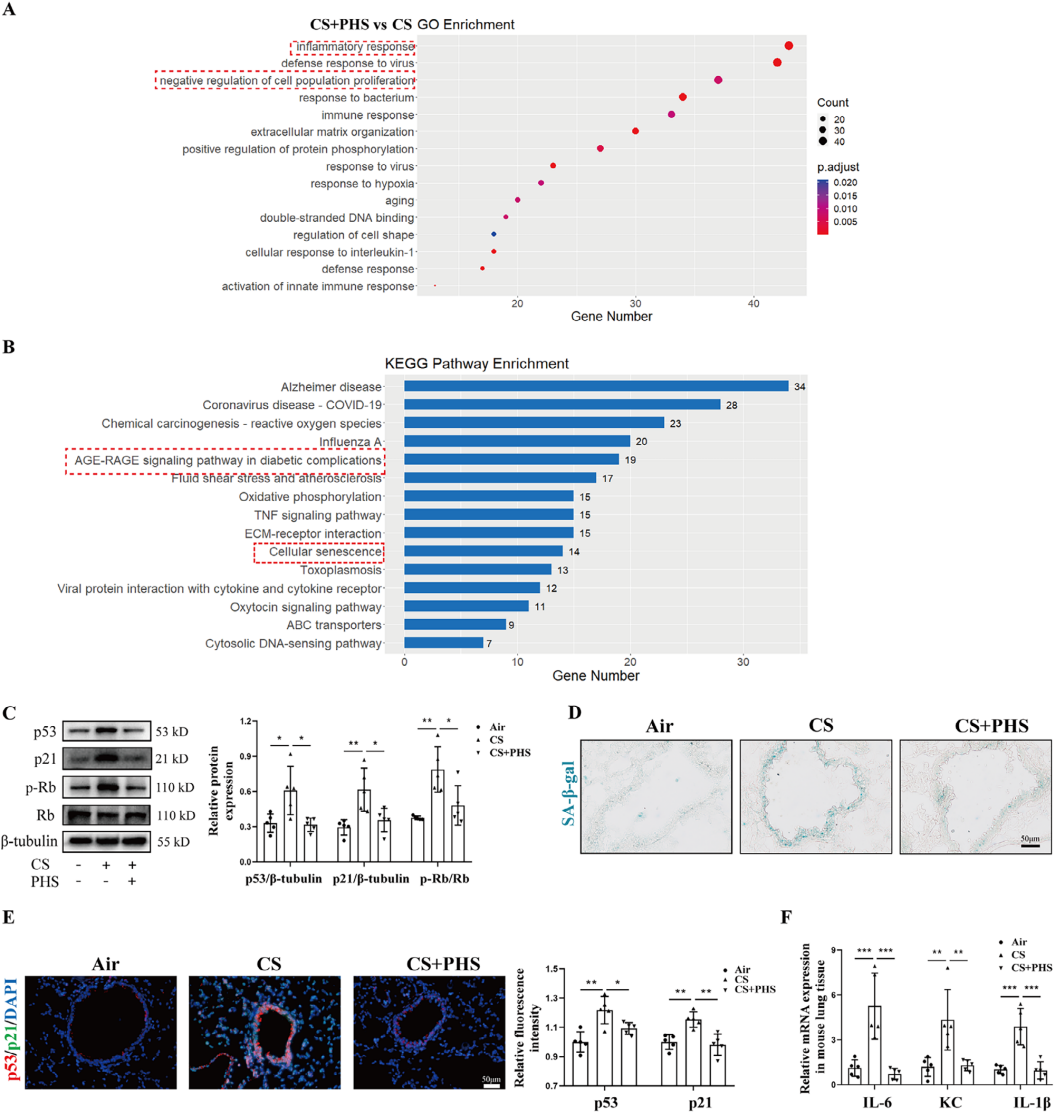
Phytosphingosine alleviated airway epithelial cell senescence in CS‐exposed mice. **(A)** RNA‐sequencing of the lung tissues from CS‐exposed mice with or without PHS treatment was performed (*n* = 3 per group). The results of biological process enrichment analysis of DEGs. **(B)** The results of KEGG pathway enrichment. **(C)** Western blot analysis of senescent proteins in mouse lung tissues, including p53, p21, and p‐Rb/Rb. **(D)** SA‐β‐gal staining for frozen lung sections of mice (staining in green indicated the positively stained area). Scale bar = 50 µm, magnification = 400x. **(E)** Representative images of co‐immunostaining of p53 and p21 in mouse lung tissues and the semi‐quantitative results. Scale bar = 50 µm, magnification = 400x. **(F)** Real‐time qPCR analysis of IL‐6, KC, and IL‐1β expressions in mouse lung tissues. Data were expressed as mean ± SD. *p*‐values were calculated using one‐way ANOVA. *p < 0.05, **p < 0.01, and ***p < 0.001 represented significant differences. CS, cigarette smoke; DEGs, differentially expressed genes; KEGG, Kyoto Encyclopedia of Genes and Genomes; PHS, phytosphingosine; SA‐β‐gal, senescence‐associated β‐galactosidase.

Subsequently, the lung tissues of mice from the CS and CS+PHS groups underwent RNA sequencing, revealing differentially expressed genes primarily associated with aging based on Gene Ontology (GO) analysis and cellular senescence according to Kyoto Encyclopedia of Genes and Genomes (KEGG) analysis (Figures 2A,B). To investigate PHS's anti‐aging properties, experiments were conducted to evaluate its impact on lung senescence in CS‐exposed mice. Western blot analysis of senescent markers showed significant upregulation of p53, p21, and p‐Rb in CS‐exposed mice compared to the Air control group, while CS+PHS mice exhibited significant downregulation of these markers (Figure 2C). Senescence‐related β‐galactosidase (SA‐β‐gal) staining indicated that PHS mitigated CS‐induced lung senescence, particularly in the bronchial epithelium (Figure 2D). Co‐immunofluorescence targeting P53 and P21 revealed that PHS treatment reduced the expression of senescent proteins in the airway epithelium induced by CS (Figure 2E). RT‐qPCR analysis of lung tissues further confirmed the beneficial effects of PHS on SASP expression, including IL‐6, KC, and IL‐1β (Figure 2F). These findings collectively support the conclusion that PHS administration can improve lung function, attenuate emphysema progression, alleviate airway inflammation, and mitigate bronchial epithelial senescence in CS‐exposed mice.

### 2.2 PHS Alleviated CSE‐Induced Cellular Senescence in Bronchial Epithelial Cells by Activating the Receptor FFAR4

**FIGURE 3:**
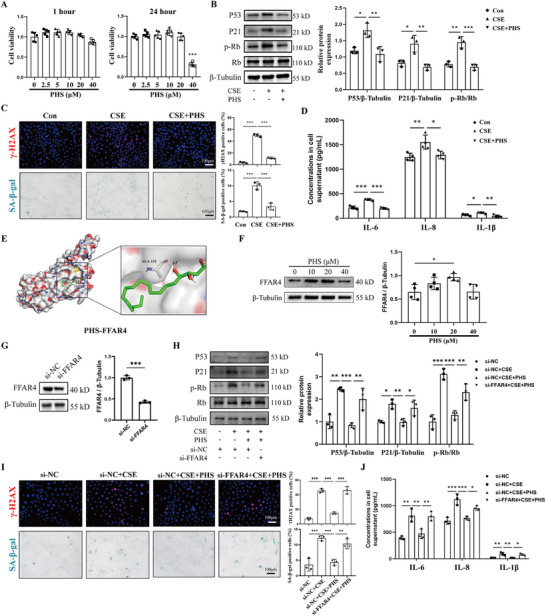
Phytosphingosine alleviated CSE‐induced cellular senescence in bronchial epithelial cells by activating FFAR4. **(A)** CCK8 results of PHS with different concentrations in HBE cells for 1 or 24 h. **(B)** Western blot analysis of P53, P21, and p‐Rb/Rb in HBE cells induced by CSE or PHS. **(C)** The representative immunofluorescent images against γ‐H2AX, representative images of SA‐β‐gal staining and their semi‐quantitative results. Scale bar = 100 µm, magnification = 200x. **(D)** ELISA results of IL‐6, IL‐8, and IL‐1β levels in the supernatant of HBE cells. **(E)** Molecular docking of PHS with FFAR4. **(F)** Western blot results of FFAR4 expressions in HBE cells treated with different concentrations of PHS. **(G)** Western blot analysis of FFAR4 in HBE cells with si‐FFAR4 treatment. **(H)** Western blot analysis of P53, P21, and p‐Rb/Rb in CSE‐exposed HBE cells with PHS or si‐FFAR4 treatment. **(I)** The representative immunofluorescent images against γ‐H2AX, representative images of SA‐β‐gal staining and their semi‐quantitative results. Scale bar = 100 µm, magnification = 200x. **(J)** ELISA results of IL‐6, IL‐8, and IL‐1β levels in the supernatant of HBE cells. Data were expressed as mean ± SD. *p*‐values were calculated using one‐way ANOVA. *p < 0.05, **p < 0.01, and ***p < 0.001 represented significant differences. CSE, cigarette smoke extract; FFAR4, free fatty acid receptor 4; HBE, human bronchial epithelial; PHS, phytosphingosine; SA‐β‐gal, senescence‐associated β‐galactosidase; siRNA, small interfering RNA.

The bronchial epithelium was found to be the primary site of senescence alleviated by PHS. To further investigate the impact of PHS, we utilized CS extract (CSE)‐treated human bronchial epithelial (HBE) cells in an in vitro model. Treatment with PHS at concentrations equal to or less than 20 µM did not show any significant toxicity on HBE cells, as indicated in Figure 3A. Subsequent analysis at 20 µM revealed that PHS downregulated senescence‐related markers P53, P21, and p‐Rb in CSE‐induced HBE cells, as shown in Figure 3B. Immunofluorescence and SA‐β‐gal staining confirmed these findings, demonstrating a lower percentage of positively stained cells in the CSE+PHS group compared to the CSE group (Figure 3C). Additionally, levels of SASP components including IL‐6, IL‐8, and IL‐1β were notably reduced in the supernatant of HBE cells in the CSE+PHS group relative to the CSE group (Figure 3D).

In a previous study, it was demonstrated that PHS binds to and activates the free fatty acid receptor 4 (FFAR4), initiating subsequent cellular processes [21]. Through molecular docking simulations, we confirmed the strong affinity between PHS and FFAR4 (Figure 3E). Our investigation revealed that treatment with 20 µM PHS significantly increased FFAR4 expression in HBE cells (Figure 3F). To further investigate the mediating role of FFAR4, we performed the recovery experiments via the knockdown of FFAR4. As shown in Figure 3G,H, expressions of the senescent proteins P53, P21, and p‐Rb were significantly increased following transfection with si‐FFAR4, relative to that in CSE‐induced HBE cells with PHS treatment. Consistent results were observed in immunofluorescence for γ‐H2AX and SA‐β‐gal staining, as well as in the release of SASP (Figure 3I,J). In summary, PHS ameliorated CSE‐stimulated cellular senescence dependent on FFAR4 activation.

### 2.3 FFAR4 was Down‐Regulated in COPD and Negatively Associated With Airway Epithelial Cell Senescence

**FIGURE 4:**
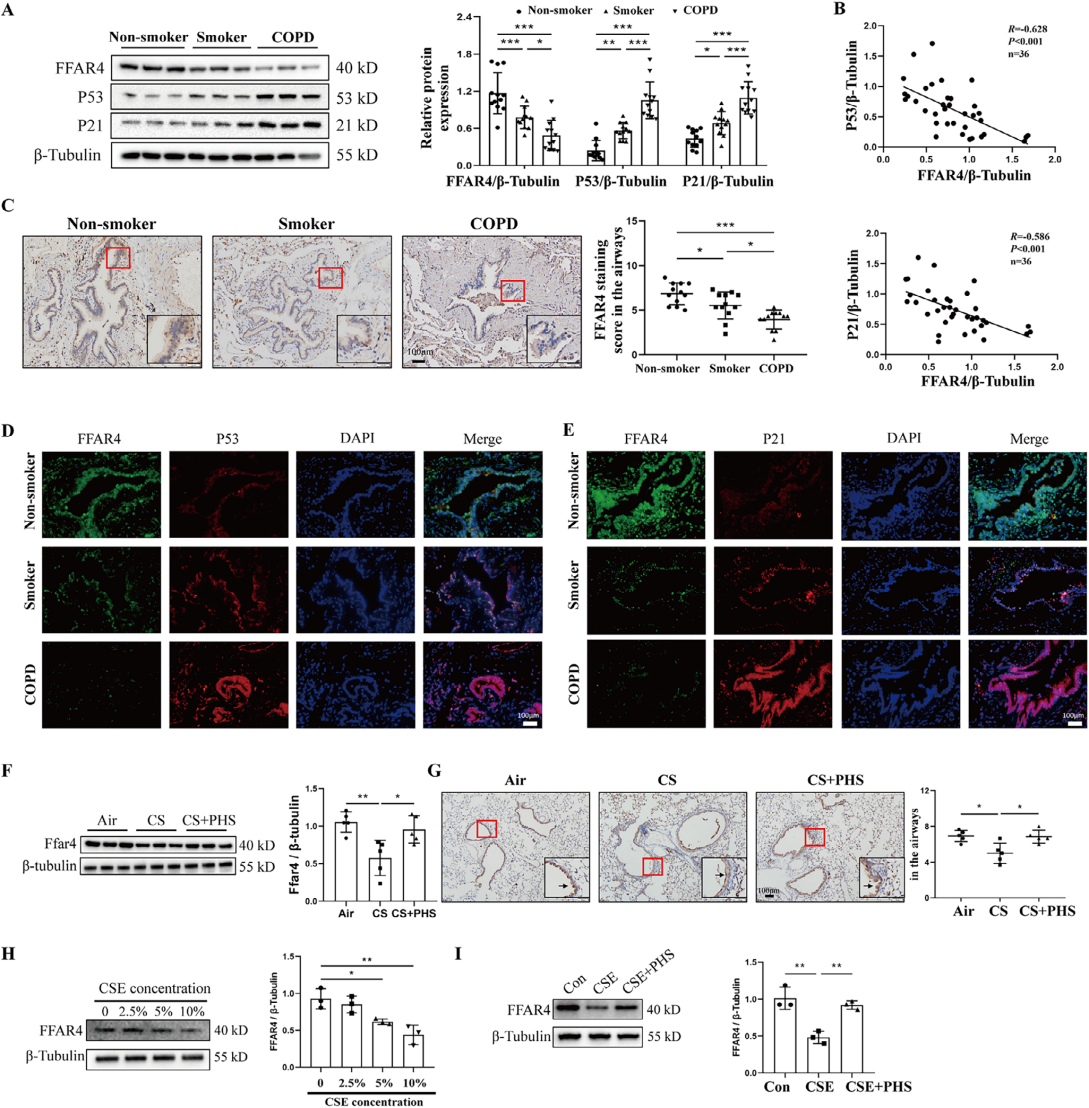
FFAR4 was downregulated in COPD and negatively associated with airway epithelial cell senescence. **(A)** Western blot analysis of FFAR4, P53, and P21 in the lung tissues from non‐smokers (*n* = 12), smokers (*n* = 12), and COPD patients (*n* = 12). **(B)** Correlation analysis between FFAR4 and P53 or P21. **(C)** The immunohistochemical staining against FFAR4 in the human lung sections and the semi‐quantitative results. Scale bar = 100 µm, magnification = 200x. **(D)** Immunofluorescent co‐staining for FFAR4 and P53 in the human lung sections. **(E)** Immunofluorescent co‐staining for FFAR4 and P21 in the human lung sections. Scale bar = 100 µm, magnification = 200x. **(F)** Western blot analysis of Ffar4 in the lung tissues from CS‐exposed mice with or without PHS. **(G)** The immunohistochemical staining against Ffar4 in the mouse lung sections and the semi‐quantitative results. The arrowheads indicated tan staining changes in the bronchial epithelium. Scale bar = 100 µm, magnification = 200x. **(H)** Western blot results of FFAR4 expressions in HBE cells treated with different concentrations of CSE. **(I)** Western blot results of FFAR4 expressions in HBE cells treated by the combination of PHS and CSE. Data were expressed as mean ± SD. *p*‐values were calculated using one‐way ANOVA. *p < 0.05, **p < 0.01, and ***p < 0.001 represented significant differences. COPD, chronic obstructive pulmonary disease; CSE, cigarette smoke extract; FFAR4, free fatty acid receptor 4; HBE, human bronchial epithelial; PHS, phytosphingosine.

Given the pivotal role of FFAR4 in PHS‐alleviated senescence, we explored the co‐expressions of FFAR4 and senescent marker P53 or P21 in the human lung specimens. Our findings revealed a progressive decline in FFAR4 expression among non‐smokers, smokers, and COPD patients, concurrent with elevated levels of P53 and P21 (Figure 4A). Furthermore, correlation analysis demonstrated a negative relationship between FFAR4 expression and senescent proteins (Figure 4B). Immunohistochemical analysis revealed predominant FFAR4 expression in the bronchial epithelium (Figure 4C). Immunofluorescence co‐staining confirmed this pattern and showed co‐expression with senescent proteins (Figure 4D,E). Western blot and immunohistochemical analyses in mouse lung tissues also indicated altered Ffar4 expression, primarily in the bronchial epithelium (Figure 4F,G). In vitro experiments further demonstrated reduced FFAR4 expression under CSE stimulation, with recovery upon PHS treatment (Figure 4H,I). To sum up, these results suggested the pivotal role of FFAR4 in the regulation of airway epithelial cell senescence in COPD.

### 2.4 Ffar4 Overexpression or Administration of Ffar4 Agonists Mitigated Lung Function Decline, Emphysema, Airway Inflammation, and Bronchial Epithelial Senescence in CS‐Exposed Mice

**FIGURE 5:**
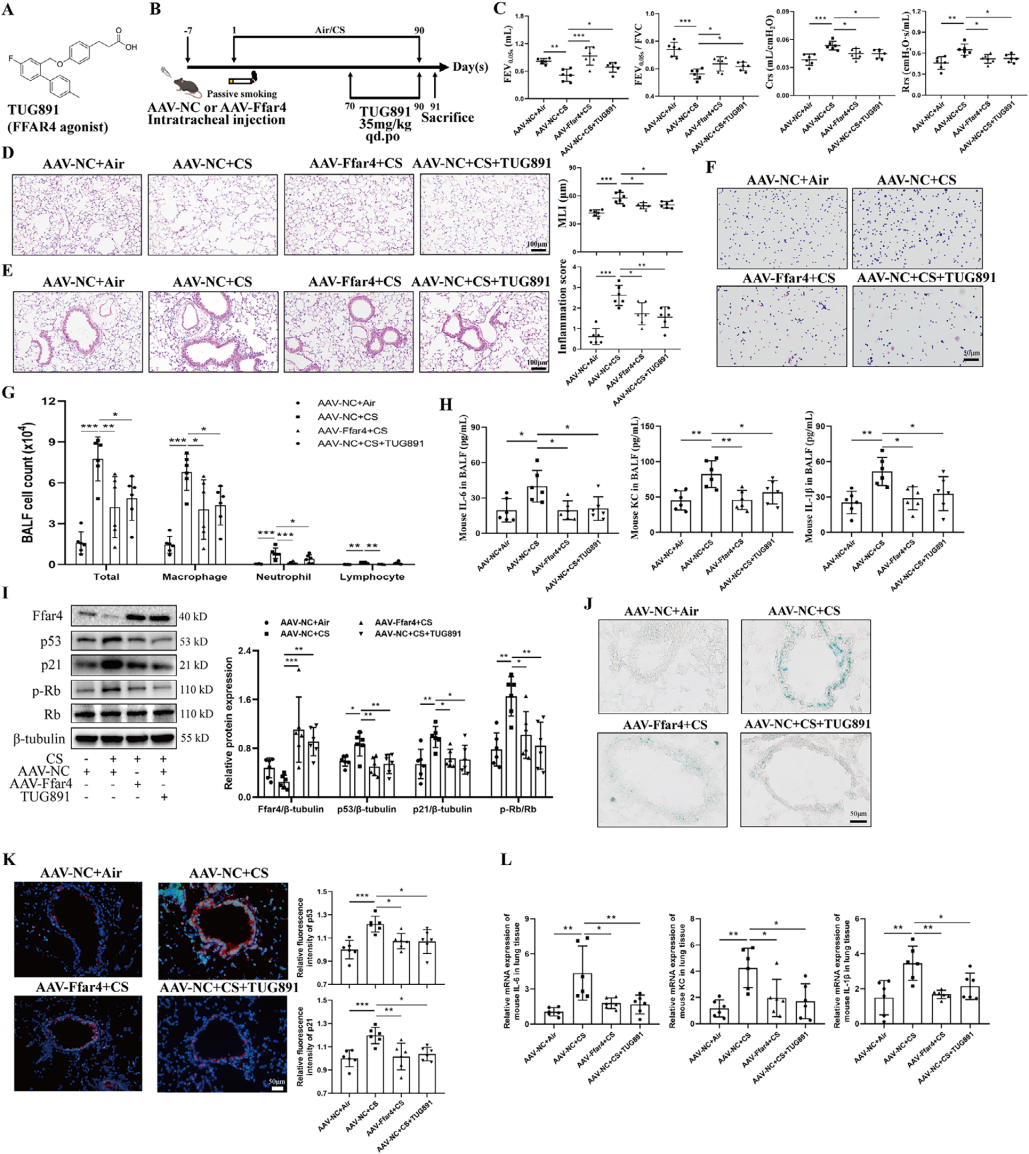
Ffar4 overexpression or administration of Ffar4 agonists mitigated lung function decline, emphysema, airway inflammation and bronchial epithelial senescence in CS‐exposed mice. **(A)** The chemical structure of TUG891. **(B)** CS‐induced COPD‐model mice and the administration methods of AAV‐Ffar4 or TUG891. **(C)** The results of lung function including FEV_0.05s_, FEV_0.05S_/FVC, Crs, and Rrs. **(D)** The representative images of HE‐stained lung sections across the alveolar area and the semi‐quantitative results of MLI indicating the emphysema extent. Scale bar = 100 µm, magnification = 200x. **(E)** The representative images of HE‐stained lung sections around the airway and the results of inflammation score. Scale bar = 100 µm, magnification = 200x. **(F)** The representative images of Liu's staining against various cells in mouse BALF. **(G)** The numbers of total cells, macrophages, neutrophils, and lymphocytes in mouse BALF. **(H)** ELISA analysis of IL‐6, KC, and IL‐1β levels in mouse BALF supernatant. **(I)** Western blot analysis of Ffar4 and senescent proteins (including p53, p21, and p‐Rb/Rb) in mouse lung tissues. **(J)** SA‐β‐gal staining for frozen lung sections of mice (green indicated the positively stained area). Scale bar = 50 µm, magnification = 400x. **(K)** Representative images of co‐immunostaining of p53 and p21 in mouse lung tissues and semi‐quantitative results. Scale bar = 50 µm, magnification = 400x. **(L)** Real‐time qPCR analysis of IL‐6, KC, and IL‐1β in mouse lung tissues. Data were expressed as mean ± SD. *p*‐values were calculated using one‐way ANOVA. *p < 0.05, **p < 0.01, and ***p < 0.001 represented significant differences. AAV, adeno‐associated virus; BALF, bronchoalveolar lavage fluid; COPD, chronic obstructive pulmonary disease; Crs, compliance of respiratory system; CS, cigarette smoke; FEV_0.05s_, forced expiratory volume in 0.05 s; Ffar4, free fatty acid receptor 4; FVC, forced vital capacity; HE, hematoxylin‐eosin; MLI, mean linear intercept; NC, negative control; Rrs, respiratory system resistance; SA‐β‐gal, senescence‐associated β‐galactosidase.

To furtherly investigate the in vivo role of the receptor FFAR4 in CS‐induced pathophysiology, we established a COPD mouse model by administering adeno‐associated virus (AAV)‐Ffar4 intratracheally or TUG891 orally (its chemical structure of TUG891 is shown in Figure 5A; the simplified diagram is shown in Figure 5B). Blood samples and H&E staining were collected to assess hepatic, renal, and cardiac function, confirming no apparent toxicity among groups (Figure S2). Pulmonary function tests revealed significant improvements in FEV_0.05_, FEV_0.05_/FVC, Crs, and Rrs in AAV‐Ffar4+CS group and AAV‐NC+CS+TUG891 group compared to AAV‐NC+CS group (Figure 5C). H&E staining indicated the alleviating effect of Ffar4 on CS‐induced emphysema, with reduced lung destruction and lower MLI in Ffar4‐overexpressing mice and TUG891‐treated mice compared to the CS group (Figure 5D). Analysis of inflammatory cell infiltration around the airways revealed that both Ffar4 overexpression and TUG891 treatment significantly decreased the number of inflammatory cells induced by CS exposure, as shown by H&E staining and immune cell classification counts in BALF (Figure 5E–G). ELISA analysis of BALF supernatant demonstrated markedly reduced levels of proinflammatory factors IL‐6, KC, and IL‐1β in the AAV‐Ffar4+CS group and AAV‐NC+CS+TUG891 group (Figure 5H).

Furthermore, we investigated the involvement of Ffar4 in pulmonary senescence using western blot analysis, SA‐β‐gal staining, and SASP assay. As shown in Figure 5I, senescent markers including p53, p21, and p‐Rb were notably downregulated in the AAV‐Ffar4+CS and AAV‐NC+CS+TUG891 groups compared to the AAV‐NC+CS group. This decrease in bronchial epithelial senescence was further confirmed by SA‐β‐gal staining (Figure 5J). Immunofluorescence results also indicated a significant decrease in P53 and P21 levels in the airway epithelium of the AAV‐Ffar4+CS and AAV‐NC+CS+TUG891 groups compared to the AAV‐NC+CS group (Figure 5K). The SASP assay corroborated these findings observed in the western blot analysis (Figure 5L). In summary, the results suggest that overexpression of Ffar4 or treatment with TUG891 can effectively mitigate the decline in lung function, emphysema, airway inflammation, and bronchial epithelial senescence induced by CS exposure in mice.

### 2.5 FFAR4 Attenuated Cellular Senescence in CSE‐Induced Bronchial Epithelial Cells

**FIGURE 6:**
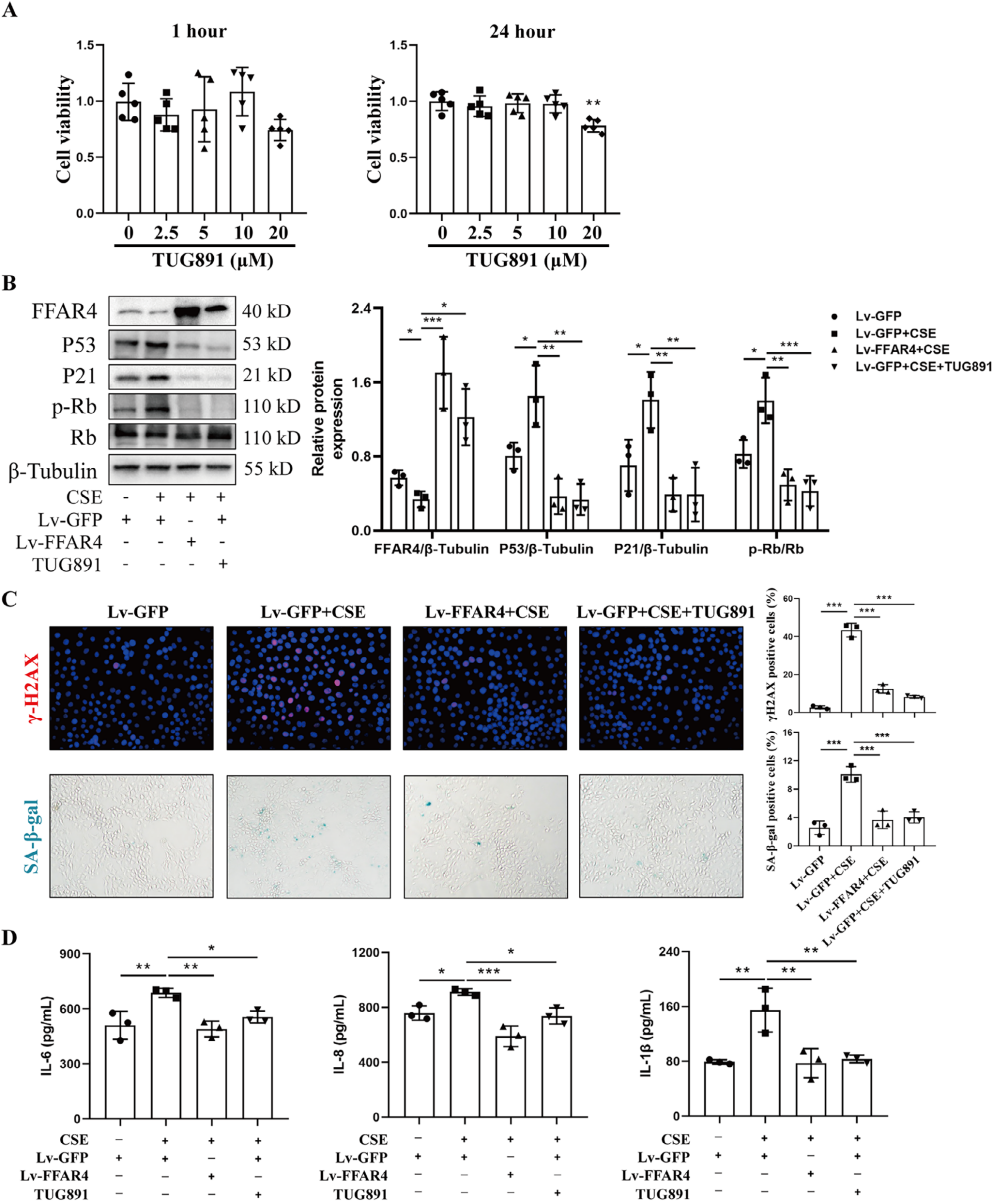
FFAR4 overexpression or administration of FFAR4 agonists attenuated cellular senescence in CSE‐induced bronchial epithelial cells. **(A)** CCK8 results of TUG891 with different concentrations in HBE cells for 1 or 24 h. **(B)** Western blot analysis of FFAR4, P53, P21, and p‐Rb/Rb in CSE‐induced HBE cells treated by Lv‐FFAR4 or TUG891. **(C)** The representative immunofluorescent images against γ‐H2AX, representative images of SA‐β‐gal staining and their semi‐quantitative results. Scale bar = 100 µm, magnification = 200x. **(D)** ELISA analysis of IL‐6, IL‐8, and IL‐1β levels in the supernatant of HBE cells. Data were expressed as mean ± SD. *p*‐values were calculated using one‐way ANOVA. *p < 0.05, **p < 0.01, and ***p < 0.001 represented significant differences. CSE, cigarette smoke extract; FFAR4, free fatty acid receptor 4; HBE, human bronchial epithelial; Lv, lentivirus; Lv‐GFP, lentivirus with empty vector carrying green fluorescent protein; SA‐β‐gal, senescence‐associated β‐galactosidase.

The role of FFAR4 in CSE‐induced senescence in HBE cells was investigated through Lv‐FFAR4 infection or TUG891 pharmacological treatment. HBE cells were exposed to varying concentrations of TUG891, with 10 µM showing no adverse effects on cell viability based on CCK8 assay results (Figure 6A). Western blot analysis confirmed increased FFAR4 expression in Lv‐FFAR4 and TUG891‐treated HBE cells (Figure 6B). Following CSE exposure, upregulation of senescent proteins P53, P21, and p‐Rb was observed, which was mitigated significantly by FFAR4 overexpression or TUG891 treatment (Figure 6B). Immunofluorescence analysis of γ‐H2AX and SA‐β‐gal staining supported these findings (Figure 6C). ELISA results for SASP detection indicated a notable reduction in IL‐6, IL‐8, and IL‐1β levels in cell supernatant upon FFAR4 overexpression and TUG891 treatment post‐CSE stimulation (Figure 6D). In conclusion, upregulation of FFAR4 or treatment with FFAR4 agonist TUG891 in HBE cells showed potential in alleviating CSE‐induced cellular senescence.

### 2.6 FFAR4 Interacted With STUB1 to Ameliorate Bronchial Cell Senescence by Modulating the Ubiquitination of P53

**FIGURE 7:**
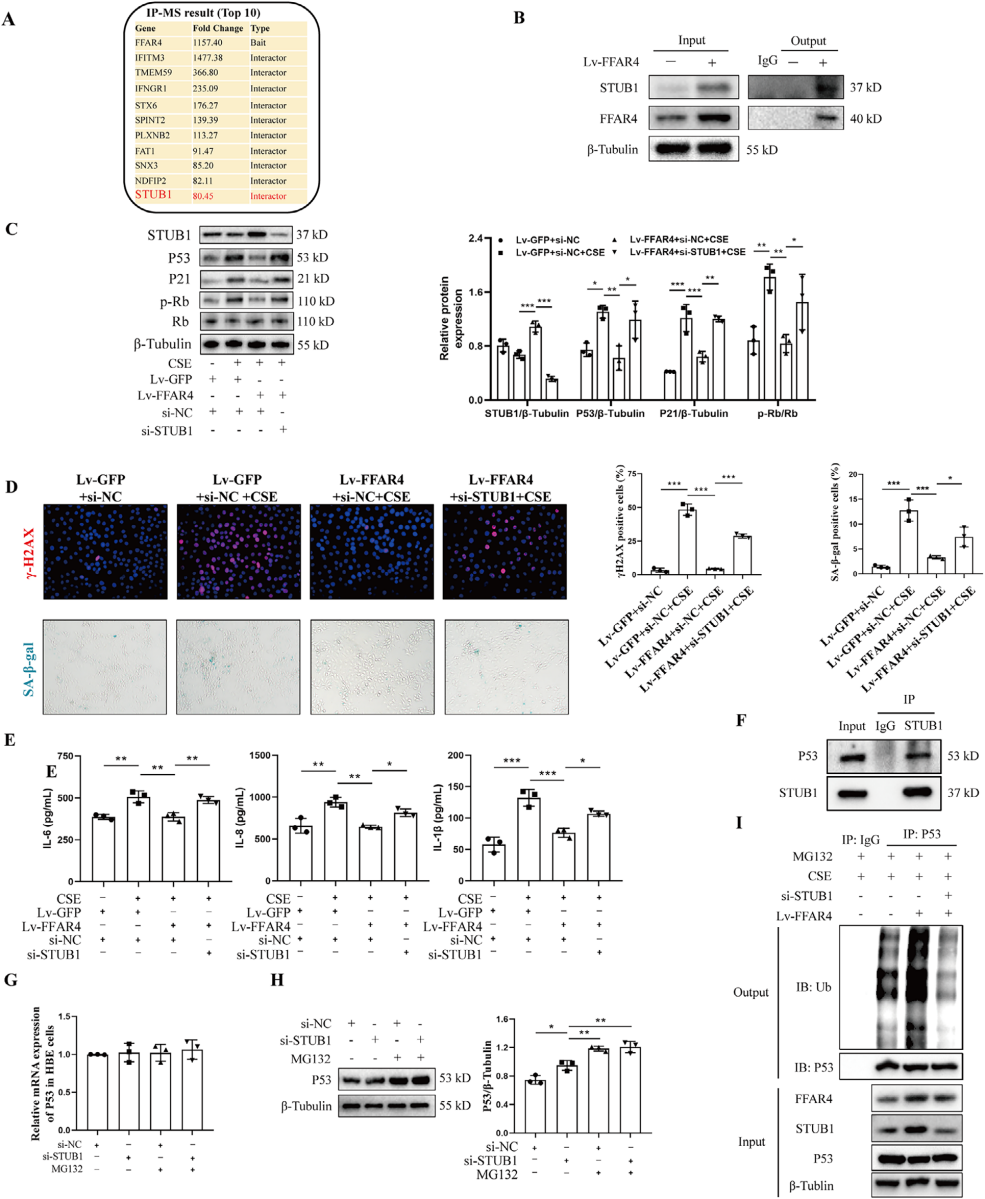
FFAR4 interacted with STUB1 to ameliorate cellular senescence in bronchial epithelial cells via enhancing the ubiquitination of P53. **(A)** Top 10 proteins baited by FFAR4 from IP/MS detection. **(B)** Co‐immunoprecipitation analysis of FFAR4 and STUB1. **(C)** Western blot analysis of STUB1, P53, P21, and p‐Rb/Rb in CSE‐induced HBE cells treated by Lv‐FFAR4 or si‐STUB1. **(D)** The representative immunofluorescent images against γ‐H2AX, representative images of SA‐β‐gal staining and their semi‐quantitative results. Scale bar = 100 µm, magnification = 200x. **(E)** ELISA analysis of IL‐6, IL‐8, and IL‐1β levels in the supernatant of HBE cells. **(F)** Co‐immunoprecipitation analysis of STUB1 and P53. **(G)** Real‐time qPCR analysis of P53 in HBE cells treated with si‐STUB1 or MG132. **(H)** Western blot analysis of P53 in HBE cells treated with si‐STUB1 or MG132. **(I)** Ubiquitination change of P53 in CSE‐induced HBE cells treated with si‐STUB1 or Lv‐FFAR4. Data were expressed as mean ± SD. *p*‐values were calculated using one‐way ANOVA. *p < 0.05, **p < 0.01, and ***p < 0.001 represented significant differences. CSE, cigarette smoke extract; FFAR4, free fatty acid receptor 4; HBE, human bronchial epithelial; IP/MS, immunoprecipitation‐mass spectrometry; Lv, lentivirus; Lv‐GFP, lentivirus with empty vector carrying green fluorescent protein; SA‐β‐gal, senescence‐associated β‐galactosidase; si, small interfering RNA; STUB1, STIP1 homology and U‐Box containing protein 1.

To explore the mechanism of FFAR4‐alleviated cellular senescence, we performed immunoprecipitation to isolate FFAR4 protein from HBE cells, followed by mass spectrometry analysis. STIP1 homology and U‐Box containing protein 1 (STUB1) was identified as a significant binding partner through an immunoprecipitation‐mass spectrometry (IP‐MS) assay (Figure 7A), and the FFAR4‐STUB1 interaction was confirmed via an IP experiment (Figure 7B). Immunofluorescence co‐staining of human and mouse lung sections revealed co‐expression of FFAR4 and STUB1 in bronchial epithelium (Figure S3A,B). Treatment with PHS was found to enhance the interaction between these two proteins (Figure S3C). Functional recovery experiments involving si‐STUB1 transfection validated the role of STUB1 in mediating FFAR4 effects. Overexpression of FFAR4 in HBE cells upregulated STUB1 expression, while si‐STUB1 transfection significantly reduced STUB1 levels (Figure 7C). Western blot analysis showed that FFAR4 overexpression in HBE cells decreased the expression of senescent proteins P53, P21, and p‐Rb induced by CSE, whereas STUB1 knockdown led to their upregulation (Figure 7C). This was supported by immunofluorescence staining for γ‐H2AX and SA‐β‐gal staining (Figure 7D). ELISA analysis for SASP indicated that FFAR4 overexpression reduced the production of IL‐6, IL‐8, and IL‐1β induced by CSE, with STUB1 knockdown reversing this effect (Figure 7E). Collectively, these results suggest that FFAR4's ability to alleviate cellular senescence is dependent on STUB1 presence.

As reported, STUB1, serving as an ubiquitinase, could affect the ubiquitination of P53, a key protein initiating cellular senescence [22, 23], though the similar mechanism in COPD has not been explored. Our IP assay validated STUB1's interaction with P53 in HBE cells (Figure 7F). Interestingly, PHS treatment could upregulate STUB1 expression and the interaction between STUB1 and P53 (Figure S4). Knocking down STUB1 augmented P53 protein levels without altering mRNA levels, and treatment with the protease inhibitor MG132 further increased P53 protein levels (Figure 7G,H), indicating STUB1's involvement in P53 degradation. To assess STUB1's impact on P53 protein degradation via ubiquitination modification, we conducted IP purification of P53 followed by detection with a ubiquitination antibody. Our results revealed that FFAR4 overexpression enhanced P53 ubiquitination while STUB1 knockdown decreased it (Figure 7I). Collectively, FFAR4 decreased the intracellular levels of P53 by interacting with STUB1 to modulate P53 ubiquitination, thereby mitigating cellular senescence in HBE cells.

## 3 Discussion

**FIGURE 8:**
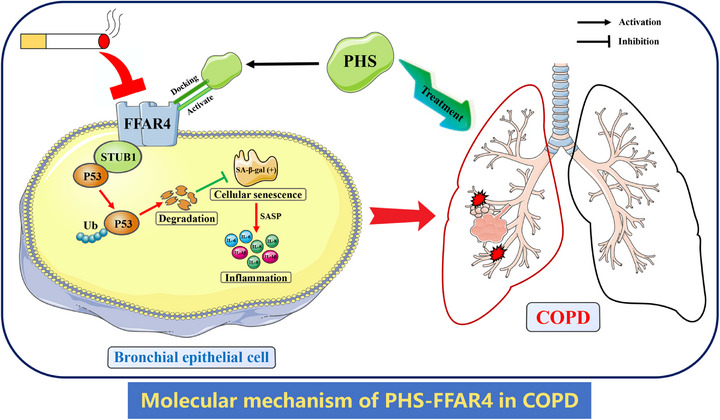
Diagram of mechanism underlying PHS‐FFAR4 regulation in CS‐induced cellular senescence of bronchial epithelial cell. Cigarette smoke inhibited FFAR4 expression and the interaction with STUB1 in bronchial epithelial cell, leading to decreased ubiquitination degradation of P53, conducing to cellular senescence and senescence‐related inflammation. Administration of phytosphingosine could combine with FFAR4 and promote its expression, furtherly improving cellular senescence and COPD progression. COPD, chronic obstructive pulmonary disease; FFAR4, free fatty acid receptor 4; PHS, phytosphingosine; SA‐β‐gal, senescence‐associated β‐galactosidase; STUB1, STIP1 homology and U‐Box containing protein 1; Ub, ubiquitin.

In this study, we investigated the therapeutic potential of PHS for COPD and elucidated its underlying molecular mechanism. Our results show that CS suppresses FFAR4 expression and its interaction with STUB1 in bronchial epithelial cells, leading to reduced ubiquitination of P53, resulting in cellular senescence and senescence‐related inflammation. Conversely, treatment with PHS mitigates cellular senescence and inflammation by binding to FFAR4 and enhancing its expression, thereby ameliorating COPD progression (Figure 8). These findings introduce PHS as a promising therapeutic candidate for COPD, with potential for future clinical applications.

The advancement of omics technology has facilitated the elucidation of the metabolic aspects of COPD through metabolomic sequencing. Metabolomic abnormalities and changes have been identified in COPD patients using various clinical specimens such as BALF, lung tissues, blood, urine, and fecal samples [24], largely attributed to chronic CS exposure. Cigarette smoking, a primary external risk factor for COPD, exerts complex effects on metabolism. Studies have shown that smokers exhibit distinct expressions of glycerophospholipids and sphingophospholipids in blood samples, correlating with worsened lung function and prognosis [25]. Similar associations have been observed for other metabolites including branched‐chain amino acids, eicosanoids, fibrinogen, and specific ions [26, 27, 28]. Additionally, passive smoking has been found to induce metabolic alterations in amino acids, phospholipids, and purines, activating inflammatory pathways and oxidative stress, thereby contributing to the progression of COPD [29, 30]. These metabolites can serve as biomarkers for representing COPD severity and predicting prognosis. Previous research has shown that the levels of phospholipids within the lungs are closely related to the presence and extent of emphysema [31]. Elevated ceramide levels in COPD patients, compared to healthy individuals, indicate increased lung tissue damage and disease severity [32]. Our team's previous study also identified metabolic disruptions in COPD lung tissues, particularly in glycerophospholipids and amino acids [14]. Further analysis of metabolomic sequencing data revealed a decrease in PHS levels within the lungs as COPD progressed, showing a strong positive correlation with lung function. This suggests PHS's potential as a biomarker for predicting COPD severity.

PHS, a sphingolipid type, is predominantly present in plants, yeast, mammalian tissues, and cells. It can be metabolized into odd‐numbered fatty acids and glycerophospholipids, influencing apoptosis, migration, and inflammation regulation [33]. Sphingolipids play a significant role in COPD pathogenesis, affecting disease initiation and progression [34]. Ceramide is a key focus in sphingolipid metabolism, and administering its downstream metabolite sphingosine‐1‐phosphate (S1P) to the lungs can counteract the proapoptotic effects of excessive ceramide and emphysema induced by CS exposure [35]. Ceramide and S1P are involved in various COPD processes, such as endothelial barrier dysfunction, oxidative stress, autophagy, and macrophage efferocytosis [34, 36, 37]. However, the role of PHS in COPD pathogenesis remains poorly characterized. Previous studies suggest that PHS may impact apoptosis and inflammation by regulating ROS‐dependent caspase 8 activation, bax translocation [38], interacting with CD300b for neutrophil recruitment [39], and activating endoplasmic reticulum stress [40]. Our study shows that PHS mitigates CS‐induced pulmonary histopathological changes, including emphysema and airway inflammation, possibly through its bioactive functions. We observed PHS's role in reducing bronchial epithelial cell senescence and SASP, contributing to improved pathology from CS exposure. A review highlighting the anti‐aging effects of the alkaline ceramidase family supports the potential role of PHS in regulating cellular senescence and aging, aligning with our study findings [41].

As reported, PHS exerts the biological function within the cells through combining and interacting with the receptor FFAR4 [42]. Here, we confirmed their via molecular docking simulation, and additionally found the favorable effect of PHS on FFAR4 expression. FFAR4, a member of G protein‐coupled receptors family, was confirmed to play a critically protective role in senescence, inflammation, and fibrosis [43, 44, 45]. Research by Yang et al. demonstrated that FFAR4 mitigates tubular epithelial cell senescence and ameliorates cisplatin‐induced acute kidney injury through the AMPK/SirT3 signaling pathway [45]. As for COPD, cellular senescence contributes significantly to disease progression. Diverse extrinsic insults such as tobacco smoking and air pollution induce DNA damage, activating p53, and downstream biological pathways [46, 47]. Evidence has established that p53 is upregulated and plays a crucial role in initiating COPD‐related airway epithelial senescence by activating p21, leading to irreversible cell cycle arrest [46]. This process enhances SASP production, hindering tissue repair and perpetuating chronic inflammation. Notably, p53 collaborates with p16INK4a pathways, sustaining senescence in COPD [47]. Consistently, our study showed that overexpressing FFAR4 or administering its agonist could effectively reduce CS‐induced increases in senescent markers like p53 and decrease SASP production, ultimately ameliorating CS‐induced senescence injury both in vivo and in vitro. These findings suggest that PHS alleviates bronchial epithelial cell senescence in response to CS exposure by interacting with FFAR4 and enhancing its expression.

Furthermore, ubiquitination's impact on cellular senescence in COPD was examined, revealing its crucial role in maintaining cellular integrity by targeting damaged proteins for degradation. However, its efficacy in clearing damaged proteins decreases with age, leading to heightened proteotoxic stress and accelerated cellular aging [48]. COPD, an age‐related disease characterized by accelerated lung aging, demonstrates impaired ubiquitin‐proteasome system function, especially in severe emphysema cases [49]. Dysregulation of ubiquitination enzymes in the lungs can enhance the expression of senescent markers such as p53 and p21, underscoring the association between ubiquitination modification and cellular senescence in COPD [50]. In the present study, we observed that FFAR4 could interact with the E3 ubiquitin‐protein ligase STUB1 and promoted its expression, where STUB1‐mediated P53 ubiquitination, thereby affecting cellular senescence. In line with our observations, Naito et al. noted that reduced STUB1 levels correlated with heightened P53 accumulation in cardiac ischemia [22].

Several limitations exist in this study. First, although the beneficial impact of PHS on COPD has been demonstrated in animal and cell studies, clinical validation is necessary to determine safe dosages and prevent adverse effects in humans. Additionally, while the therapeutic potential of both PHS and TUG891 has been identified, it remains unclear whether PHS surpasses TUG891 in its ability to restrain COPD progression, necessitating further comprehensive investigation. Furthermore, the effects of PHS on key biological processes in COPD, such as airway remodeling, are still under investigation. Apart from CS, environmental pollutants like PM2.5 and coal tar can also induce airway epithelial senescence through oxidative DNA damage and inflammation [51, 52]. Given that PHS attenuates senescence via the FFAR4/STUB1/P53 pathway, which is involved in responding to various airborne toxins, it is plausible that its protective effects may extend to these exposures, although confirmation through studies using PM2.5 or coal‐derived extracts is needed. Moreover, the role of PHS in CS‐induced macrophage senescence requires further investigation. Finally, aside from ubiquitination modification, other mechanisms governing PHS/FFAR4‐mediated improvement of bronchial epithelial cell senescence need further exploration.

## 4 Conclusions

This study highlighted PHS as a promising therapeutic candidate for COPD through modulation of the FFAR4/STUB1/P53 signaling axis. PHS effectively mitigated CS‐induced bronchial epithelial cell senescence, emphysema, airway inflammation, and lung dysfunction by upregulating FFAR4 receptor expression and activation. Mechanistically, FFAR4 interacted with STUB1 to facilitate P53 ubiquitination and degradation, thus inhibiting cellular senescence. These findings underscore PHS's potential in attenuating COPD progression and reveal a novel molecular pathway for clinical interventions. Further research is needed to validate PHS's efficacy and safety in clinical settings.

## 5 Materials and Methods

### 5.1 Secondary Analysis for Metabolomic Data

Based on untargeted metabolomic data from our previous study on COPD lung tissues [14], a secondary analysis was conducted to extract differentially expressed metabolites and baseline characteristics of the corresponding subjects. A heatmap of the top 20 metabolites showing the most significant differential expression was generated using R (version 4.3.1) with the R packages “pheatmap” and “ggplot2.” COPD cases were categorized into three groups based on the GOLD grading system: mild (FEV1% predicted > 80%), moderate (FEV1% predicted ranging from 50% to 80%), and severe (FEV1% predicted < 50%).

### 5.2 Reagents and Antibodies

PHS, TUG891 (a compound serving as a FFAR4 agonist), and MG132 were sourced from MedChem Express (Monmouth Junction, NJ, USA). The preparation of CSE was conducted using the methods described previously [53]. ELISA kits for mouse IL‐6, KC, and IL‐1β, as well as human IL‐6, IL‐8, and IL‐1β, were procured from R&D Systems (Minneapolis, MN, USA). Magnetic beads were sourced from Cell Signaling Technology (Danvers, MA, USA). Lipofectamine 3000 was obtained from Invitrogen (Carlsbad, CA, USA). Antibody against β‐tubulin, P53, P21, p‐Rb, Rb, γ‐H2AX, Flag, IgG, STUB1, and ubiquitin were purchased from Proteintech (Wuhan, China), while the FFAR4 antibody was acquired from Santa Cruz Biotechnology (CA, USA). SA‐β‐gal staining kit was procured from Solarbio (Beijing, China). CCK8 kit was obtained from ABclonal (Wuhan, China).

### 5.3 Mouse Model

The wild‐type (WT) mice, aged 6–8 weeks, employed in the experiment were acquired from Shulaibao in Wuhan, China, and were housed in specific pathogen‐free facilities at Tongji Hospital. To induce COPD in mice, a model was established by exposing them to CS daily in a chamber for 3 months (10 cigarettes/45 min/session, 4 sessions per day). The chamber conditions were maintained at carbon dioxide levels below 0.2%, oxygen levels above 18%, and smoke particulate matter concentrations between 300 and 500 mg/m^3^. According to administration of PHS, mice were grouped into Air, CS, and CS+PHS (*n* = 5 per group). In the CS+PHS group, mice received PHS orally at a dose of 25 mg/kg daily during the final 3 weeks of CS exposure. According to the administration of AAV or TUG891, mice were grouped into AAV‐NC+Air, AAV‐NC+CS, AAV‐Ffar4+CS, and AAV‐NC+CS+TUG891 (*n* = 6 per group). In specific, AAV‐NC or AAV‐Ffar4 (OBiO, Biotechnology) was delivered intratracheally to mice 2 weeks prior to CS exposure, while TUG891 (35 mg/kg) was orally administered to mice during the last 3 weeks of CS modeling, with the remaining three groups receiving an equivalent volume of saline orally. Upon completing the CS modeling, mice were anesthetized with a 1% pentobarbital intraperitoneal injection. The respiratory function was evaluated using the FlexiVent system (SCIREQ, Canada), recording vital metrics such as FEV_0.05s_, FVC, Crs, and Rrs. BALF was collected for cell counting using Liu's staining method (Baso, Zhuhai, China), along with subsequent analysis of inflammatory cytokines. The left lung was fixed in 4% paraformaldehyde and embedded in paraffin for histological staining, while the right lungs were stored at −80°C for further analysis. All animal procedures were conducted by ethical guidelines and were approved by the Animal Care and Use Committee of Tongji Hospital (TJH‐202105013).

### 5.4 Clinical Subjects

Lung tissues and clinical data were collected from participants at Tongji Hospital in Wuhan, China, between 2021 and 2024. Participants were classified into three groups based on smoking status and pulmonary function: non‐smokers, smokers, and those with COPD diagnosed according to GOLD 2021 criteria. Control groups consisted of non‐smokers and smokers matched for age and sex without COPD. Exclusion criteria included patients with other significant lung diseases or recent use of inhaled or oral steroids. Baseline characteristics of all participants are detailed in Table S1. The study received approval from the Tongji Hospital Ethics Committee (TJ‐IRB20210346), and written informed consent was obtained from all participants before enrollment.

### 5.5 Cell Culture and Treatment

HBE cells (HBE135‐E6/E7, ATCC, CRL‐2741) were cultured in RPMI 1640 medium supplemented with 10% fetal bovine serum at 37°C and 5% CO_2_. The cells were treated with PHS, CSE, or TUG891 at varying concentrations. Lentivirus overexpressing FFAR4 from OBiO Biotechnology (Shanghai, China) was used to infect HBE cells, or small interfering RNA (siRNA) targeting FFAR4 (5′‐GGCCUUCACAUUUGCUAAUTT‐3′) or STUB1 (5′‐AGG​CCA​AGC​ACGACA​AGU​A‐3′) from RiboBio (Guangzhou, China) was transfected using lipofectamine 3000. Western blot analysis was performed to confirm the efficacy of overexpression or knockdown. Subsequently, 10 µM of MG132 was added to the HBE cells to investigate P53 degradation via the proteasome pathway.

### 5.6 Histological and Immunostaining Analysis

Mouse lung sections were stained with H&E for pathological evaluation. Five random fields were photographed per HE‐stained section, and evaluations of peribronchial inflammation (indicative of airway inflammation) and MLI (indicating emphysema) were conducted following established methods [9]. In specific, peribronchial inflammation severity was assessed using a semi‐quantitative grading system adapted from prior methodology [54], with scores defined as follows: 0 (no pathology), 1 (minimal cellular infiltration), 2 (inflammatory cell layer ≤ 1 cell depth), 3 (inflammatory cell layer two to four cells deep), and 4 (inflammatory cell layer > 4 cells deep). Immunohistochemistry involved incubating human or mouse lung sections with an anti‐FFAR4 antibody (1:200, Santa Cruz Biotechnology, CA, USA) according to the manufacturer's instructions. FFAR4 immunostaining was quantified using an established semi‐quantitative scoring protocol [54]. Two observers independently assessed staining patterns, with final scores derived from the product of intensity (0: none; 1: faint yellow; 2: moderate brown; 3: intense dark brown) and extent (1: ≤ 25% positive cells; 2: 26%–50%; 3: 51%–75%; 4: ≥ 76% positivity) across airway‐adjacent regions. Immunofluorescence analysis included incubation with anti‐FFAR4 (1:50, Santa Cruz Biotechnology, CA, USA), anti‐P53 (1:100, Proteintech), anti‐P21 (1:100, Proteintech), or anti‐STUB1 (1:100, Proteintech) antibodies on human and mouse lung sections, with the semi‐quantitative analysis conducted by ImageJ. The average fluorescence intensity was equal to the total fluorescence intensity divided by the area. The relative fluorescence intensity was normalized according to the average fluorescence intensity of the control group.

### 5.7 ELISA

IL‐6, KC, and IL‐1β levels in mouse BALF, and IL‐6, IL‐8, and IL‐1β levels in HBE supernatants were measured using ELISA kits following the manufacturer's protocols.

### 5.8 Western Blot

RIPA lysis was employed for protein extraction from lung tissues or cells, followed by loading the proteins onto a gel mixed with loading buffer. The gel was then transferred to a polyvinylidene fluoride membrane and incubated overnight at 4°C with primary antibodies, followed by incubation with secondary antibodies the next day. Visualization of the membrane was achieved using the chemiluminescence procedure (Bio‐Rad, CA, USA), with quantification of band intensity conducted using ImageJ.

### 5.9 SA‐β‐gal Assay

Cryopreserved lung slices or HBE cells were treated with the SA‐β‐gal staining kit according to the manufacturer's instructions. The procedure involved immersing the samples in a fixing agent for 15 min, followed by overnight incubation with the SA‐β‐gal staining solution at 37°C. Visualization was carried out using a Nikon Spot imaging apparatus, capturing three randomly selected fields per specimen to assess the ratio of cells displaying positive staining.

### 5.10 Real‐Time qPCR

Total RNA isolated from HBE cells and lung tissues using Trizol was reverse transcribed into cDNA and amplified by RT‐qPCR. Relative gene expression was calculated by 2^−△△Ct^ formulate with β‐actin as endogenous control. Primer sequences are in Table S2.

### 5.11 Evaluation of Liver, Renal, and Cardiac Function

Liver, renal, and cardiac functions were assessed through the detecting the serum levels for ALT, AST, BUN, CR, LDH, and CK at Baiqiandu Technology Co., Ltd (Wuhan, China). Histopathological changes in mouse liver, kidney, and heart sections were assessed through H&E staining.

### 5.12 RNA Sequencing and Data Process

Total RNA was isolated and extracted from the mouse lung tissues in CS+PHS and CS group. RNA sequencing was conducted at Wuhan Benagen Technology Co., Ltd (Wuhan, China). As mentioned before, DEGs were identified as per the parameters of |logFC| > 1 and *p*‐value < 0.05. GO analysis and Kyoto Encyclopedia of Genes and Genomes (KEGG) pathway enrichment for the DEGs was performed using R (version 4.3.1).

### 5.13 CCK8

Cells in 96‐well plates were incubated with different concentrations of PHS or TUG891. Subsequently, 10 µL of CCK‐8 reagent was added, and the absorbance was measured at 450 nm using a microplate reader.

### 5.14 γ‐H2AX Immunofluorescence Staining

After fixation with 4% paraformaldehyde, HBE cells underwent a 1‐h blocking step. Subsequently, they were subjected to anti‐γ‐H2AX antibodies at 4°C overnight, followed by treatment with secondary antibodies for 1 h at room temperature. Nuclei were then stained with DAPI solution, and images were taken using an Olympus BX51 fluorescence microscope system (Japan). The enumeration of positively stained cells was performed using ImageJ.

### 5.15 Molecular Docking

The 3D structure of the FFAR4 protein was retrieved from the RCSB Protein Data Bank (http://www.rcsb.org/) and prepared using AutoDock software. The PHS compound was obtained from PubChem (https://pubchem.ncbi.nlm.nih.gov/) and underwent similar processing. Molecular docking was conducted via AutoDock Vina, with binding affinities categorized as spontaneous (< 0 kcal/mol), good (< −4.25 kcal/mol), or strong (< −7 kcal/mol). Subsequently, the docking outcomes were visualized using PyMol2.5.

### 5.16 Immunoprecipitation Assay (IP)

Cells were lysed with NP40, and incubated with anti‐IgG, anti‐Flag, or anti‐P53 and magnetic beads with rotation overnight at 4°C. The proteins were then eluted from the beads and subjected to western blot.

### 5.17 Mass Spectrometry Detection

FFAR4 protein samples enriched by immunoprecipitation were forwarded to SpecAlly Life (Wuhan, China) for subsequent mass spectrometry analysis utilizing an Orbitrap mass spectrometer. The acquired mass spectrometry data underwent processing using Proteome Discoverer (Thermo Fisher Scientific, USA).

## 6 Statistical Analysis

Data were displayed as mean ± standard deviation, unless specified otherwise, and were compared statistically via one‐way ANOVA. Correlations were evaluated using Pearson's correlation method. Statistical processing and visualization were conducted with GraphPad Prism version 8 (California, USA). A *p* value of less than 0.05 was deemed to indicate significance.

## Author Contributions

Yuan Zhan: data curation, formal analysis, software, writing – original draft, visualization, and conceptualization. Zhesong Deng: data curation, formal analysis, software, writing – original draft, and investigation. Ruonan Yang: data curation, formal analysis, and investigation. Shanshan Chen: data curation and formal analysis. Jiaheng Zhang: formal analysis and investigation. Yating Zhang: formal analysis. Hao Fu: formal analysis. Qian Huang: formal analysis. Yiya Gu: Formal analysis. Zhilin Zeng: Formal analysis. Jinkun Chen:  Formal analysis and visualization. Jixian Zhang: writing–review and editing. Jixing Wu: writing–review and editing and resources. Jungang Xie: writing–review and editing, resources, funding acquisition, and conceptualization. All authors have read and approved the final manuscript.

## Conflicts of Interest

The authors declare no conflicts of interest.

## Ethics Statement

The study was granted approval by the Tongji Hospital Ethics Committee (TJ‐IRB20210346), and written informed consent was obtained from all participants prior to their involvement in the study. All animal procedures adhered to the ethical guidelines and were approved by the Animal Care and Use Committee of Tongji Hospital (TJH‐202105013).

## Supporting information




**Table S1**. Clinical characteristics of study subjects. **Table S2**. Primer information for qPCR. **Figure S1**. The toxic effects of phytosphingosine on the mice. **Figure S2**. The toxic effects of AAV‐Ffar4 or TUG891 on the mice. **Figure S3**. PHS promoted the co‐expression of FFAR4 and STUB1 in the airway epithelial cells. **Figure S4**. PHS promoted the STUB1 expression and combination with P53 in the airway epithelial cells.

## Data Availability

The datasets used and/or analyzed during the current study are available from the corresponding author on reasonable request.
