# Correction to “Phytosphingosine Alleviates Cigarette Smoke‐Induced Bronchial Epithelial Cell Senescence in Chronic Obstructive Pulmonary Disease by Targeting the Free Fatty Acid Receptor 4”

**DOI:** 10.1002/mco2.70501

**Published:** 2025-11-20

Y. Zhan, Z. Deng, R. Yang, et al., “Phytosphingosine Alleviates Cigarette Smoke‐Induced Bronchial Epithelial Cell Senescence in Chronic Obstructive Pulmonary Disease by Targeting the Free Fatty Acid Receptor 4,” *MedComm* 6, no. 9 (2025): e70345. https://doi.org/10.1002/mco2.70345

In the process of checking the raw data [1], the authors noticed that the representative HE‐stained image in the Air group of Figure 1H was inadvertently misplaced, which needed to be corrected after the online publication of the article. The correct Figure 1H should be shown as below. The authors apologize for these oversights and declare that this correction does not affect the description, interpretation, or conclusions detailed in the original manuscript.

**FIGURE 1:**
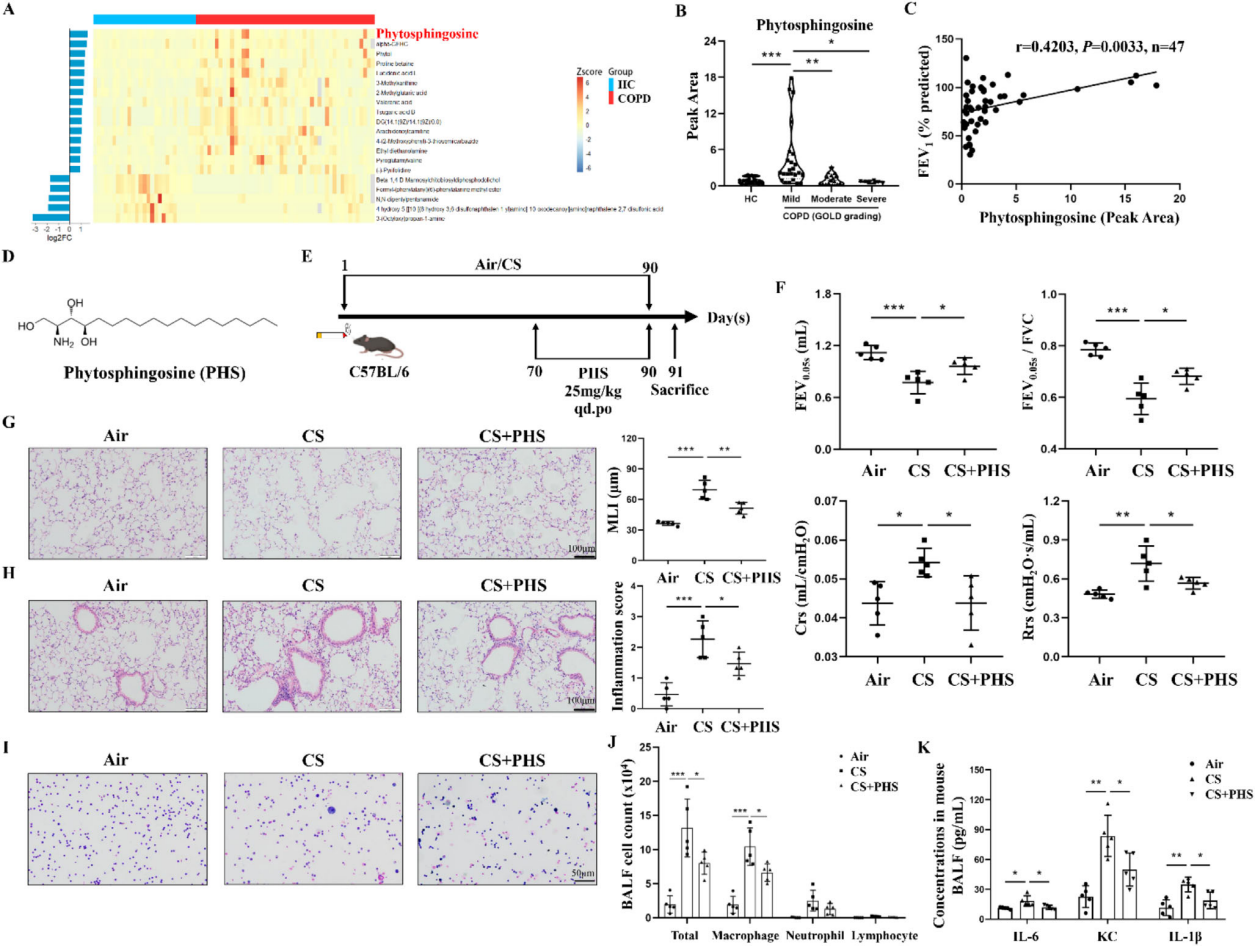
Phytosphingosine improved lung dysfunction, emphysema, airway inflammation in CS‐exposed mice. (A) Heat map of top 20 differential metabolites from the metabolomics result of human lung tissues, including 47 COPD patients and 27 controls. (B) PHS levels of lung tissues among different GOLD‐grading groups. (C) Correlation analysis of PHS and lung function in COPD patients. (D) The chemical structure of phytosphingosine. (E) CS‐induced COPD‐model mice and the method of PHS delivery. (F) The results of lung function including FEV_0.05s_, FEV_0.05s_/FVC, Crs, and Rrs. (G) The representative images of HE‐stained lung sections across the alveolar area and the semi‐quantitative results of MLI indicating the emphysema extent. Scale bar = 100 µm, magnification = 200x. (H) The representative images of HE‐stained lung sections around the airway and the results of inflammation score. Scale bar = 100 µm, magnification = 200x. (I) The representative images of Liu's staining against various cells in mouse BALF. (J) The numbers of total cells, macrophages, neutrophils, and lymphocytes in mouse BALF. (K) ELISA results of IL‐6, KC, and IL‐1β levels in BALF supernatant. Data were expressed as mean ± SD. *p*‐values were calculated using one‐way ANOVA. *p < 0.05, **p < 0.01, and ***p < 0.001 represented significant differences. BALF, bronchoalveolar lavage fluid; COPD, chronic obstructive pulmonary disease; Crs, compliance of respiratory system; CS, cigarette smoke; FEV1, forced expiratory volume in 1 s; FEV_0.05s_, forced expiratory volume in 0.05 s; FVC, forced vital capacity; GOLD, Global Initiative for Objective Assessment of Lung Disease; HC, healthy control; HE, hematoxylin‐eosin; MLI, mean linear intercept; PHS, phytosphingosine; Rrs, respiratory system resistance.

## References

[1] Y. Zhan , Z. Deng , R. Yang , et al., “Phytosphingosine Alleviates Cigarette Smoke‐Induced Bronchial Epithelial Cell Senescence in Chronic Obstructive Pulmonary Disease by Targeting the Free Fatty Acid Receptor 4,” MedComm 6, no. 9 (2025): e70345. 40895192 10.1002/mco2.70345 PMC12394999

