# Supplementary material for: Phytosphingosine Alleviates Cigarette Smoke‐Induced Bronchial Epithelial Cell Senescence in Chronic Obstructive Pulmonary Disease by Targeting the Free Fatty Acid Receptor 4
Source: MedComm (2020). 2025 Aug 29;6(9):e70345. doi: 10.1002/mco2.70345 (PMC12394999; doi:10.1002/mco2.70345)
Supplement: Supplementary file 1 — Table S1. Clinical characteristics of study subjects. Table S2. Primer information for qPCR. Figure S1. The toxic effects of phytosphingosine on the mice. Figure S2. The toxic effects of AAV‐Ffar4 or TUG891 on the mice. Figure S3. PHS promoted the co‐expression of FFAR4 and STUB1 in the airway epithelial cells. Figure S4. PHS promoted the STUB1 expression and combination with P53 in the airway epithelial cells. [file MCO2-6-e70345-s001.docx]

**Phytosphingosine alleviates cigarette smoke-induced bronchial epithelial cell senescence in chronic obstructive pulmonary disease by targeting the free fatty acid receptor 4**

**Running title: Therapeutic role of phytosphingosine in COPD**

Yuan Zhan^1,2#^, Zhesong Deng^1#^, Ruonan Yang^1^, Shanshan Chen^1^, Jiaheng Zhang^1^, Yating Zhang^1^, Hao Fu^1^, Qian Huang^1^, Yiya Gu^1^, Zhilin Zeng^3^, Jinkun Chen^4^, Jixian Zhang^5^, Jixing Wu^1^*, Jungang Xie^1^*

^1^ Department of Respiratory and Critical Care Medicine, National Clinical Research Center of Respiratory Disease, Key Laboratory of Pulmonary Diseases of Health Ministry, Tongji Hospital, Tongji Medical College, Huazhong University of Science and Technology, Wuhan, Hubei, China

^2^ Department of Respiratory and Critical Care Medicine, The First Affiliated Hospital of Chongqing Medical University, Chongqing, China

^3^ Department and Institute of Infectious Diseases, Tongji Hospital, Tongji Medical College and State Key Laboratory for Diagnosis and Treatment of Severe Zoonotic Infectious Diseases, Huazhong University of Science and Technology, Wuhan, China

^4^ Lawrence Bloomberg Faculty of Nursing, University of Toronto, 155 College Street, Suite 130, Toronto, ON, M5T 1P8

^5^ Department of Respiratory and Critical Care Medicine, Hubei Provincial Hospital of Integrated Chinese & Western Medicine, Wuhan, Hubei, China

^#^ Yuan Zhan and Zhesong Deng contributed equally to this study

* Corresponding authors:

Jungang Xie (E-mail: [xiejjgg@hotmail.com](mailto:xiejjgg@hotmail.com), Tel: +8613986299271) and Jixing Wu (E-mail: [wujixing2019@126.com](mailto:wujixing2019@126.com), Tel: +8615972929137)

Department of Respiratory and Critical Care Medicine, National Clinical Research Center of Respiratory Disease, Key Laboratory of Pulmonary Diseases of Health Ministry, Tongji Hospital, Tongji Medical College, Huazhong University of Science and Technology, Wuhan, Hubei, 430030, China

**Supplementary Material**

**Table S1** Clinical characteristics of study subjects.

**Table S2** Primer information for qPCR.

**Figure S1.** The toxic effects of phytosphingosine on the mice.

**Figure S2.** The toxic effects of AAV-Ffar4 or TUG891 on the mice.

**Figure S3.** PHS promoted the co-expression of FFAR4 and STUB1 in the airway epithelial cells.

**Figure S4.** PHS promoted the STUB1 expression and combination with P53 in the airway epithelial cells.

**Table S1** Clinical characteristics of study subjects

|  | **Non-smoker**  (n=12) | **Smoker**  (n=12) | **COPD**  (n=12) |
| --- | --- | --- | --- |
| **Age** | 59.8 (5.7) | 60.2 (7.8) | 64.5 (8.8) |
| **Gender (M/F)** | 6/6 | 6/6 | 7/5 |
| **BMI (kg/m^2^)** | 24.4 (2.9) | 23.5 (2.0) | 22.4 (2.6) |
| **Smoking (pack-yrs)** | 0 | 36.2 (20.9)* | 32.0 (19.2)* |
| **FEV1% predicted** | 111.6 (12.4) | 97.4 (18.3) | 79.9 (14.7)* |
| **FEV1/FVC** | 80.0 (4.81) | 78.5 (7.7) | 60.3 (6.2)*^#^ |

Data are expressed as mean (SD). **P*<0.05 vs subjects in Non-smoker group. ^#^*P*<0.05 vs subjects in Smoker group. COPD, chronic obstructive pulmonary disease; M/F, male/female; BMI, body mass index; FVC, forced vital capacity; FEV1, forced expiratory volume in one second.

**Table S2** Primer information for qPCR

|  | **Primer pairs (5’-3’)** |
| --- | --- |
| Mouse IL-6 | TAGTCCTTCCTACCCCAATTTCC |
|  | TTGGTCCTTAGCCACTCCTTC |
| Mouse KC | GCTTGAAGGTGTTGCCCTCAG |
|  | AAGCCTCGCGACCATTCTTG |
| Mouse IL-1β | GAAATGCCACCTTTTGACAGTG |
|  | TGGATGCTCTCATCAGGACAG |
| Mouse β-actin | AGAAAATCTGGCACCACACCT |
| Human P53  Human β-Actin | GATAGCACAGCCTGGATAGCA  CCTCAGCATCTTATCCGAGTGG  TGGATGGTGGTACAGTCAGAGC  CACCATTGGCAATGAGCGGTTC AGGTCTTTGCGGATGTCCACGT |


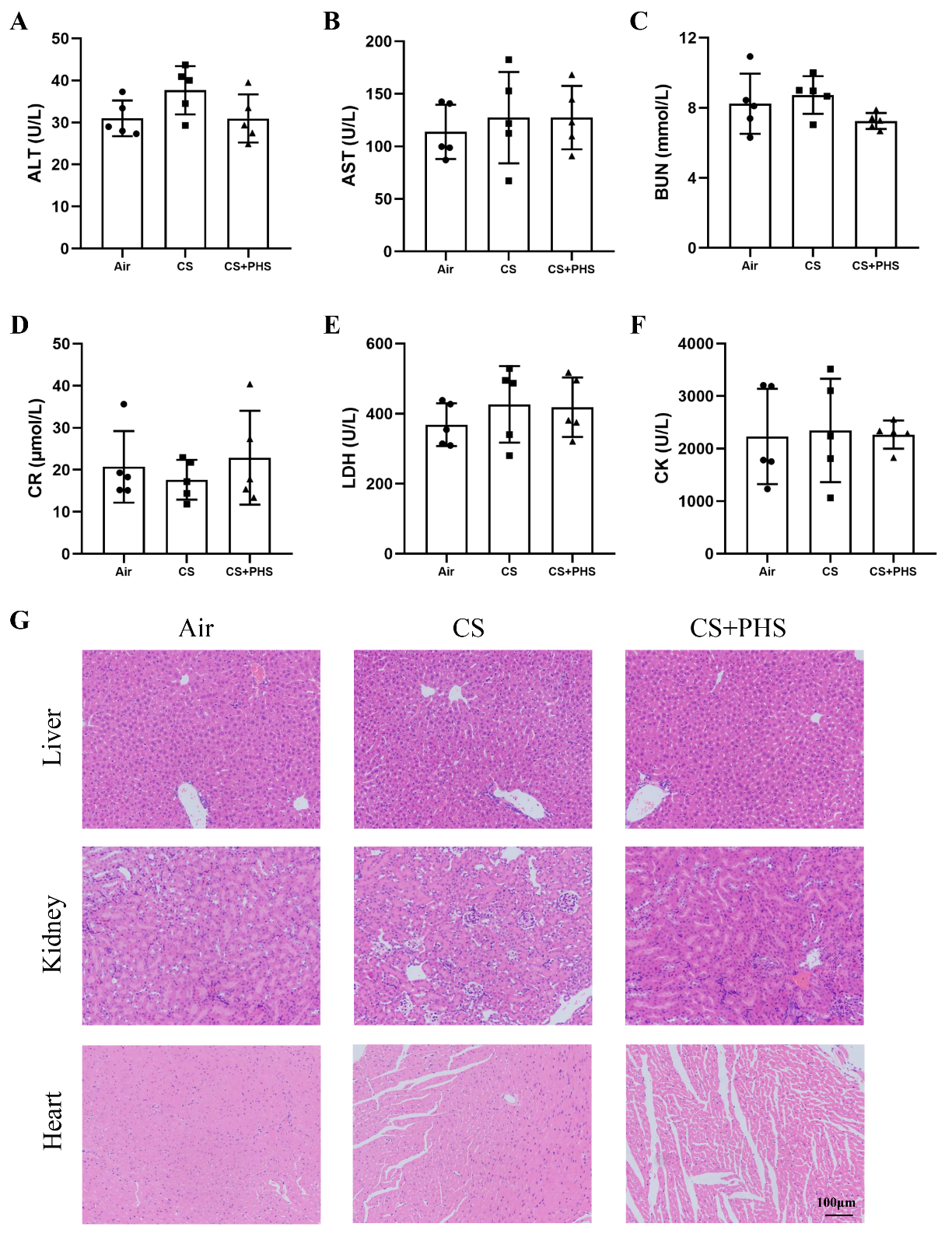


**Figure S1.** The toxic effects of phytosphingosine on the mice.

**(A, B)** Liver function. **(C, D)** Renal function. **(E, F)** Cardiac function. **(G)** The representative images of HE staining in the liver, kidney and heart tissues. Scale bar = 100μm, magnification = x 200. Data were expressed as mean ± SD. P-values were calculated using one-way ANOVA. CS, cigarette smoke; PHS, phytosphingosine; ALT, alanine aminotransferase; AST, aspartate aminotransferase; BUN, Blood Urea Nitrogen; CR, creatinine; LDH, lactate dehydrogenase; CK, creatine kinase.


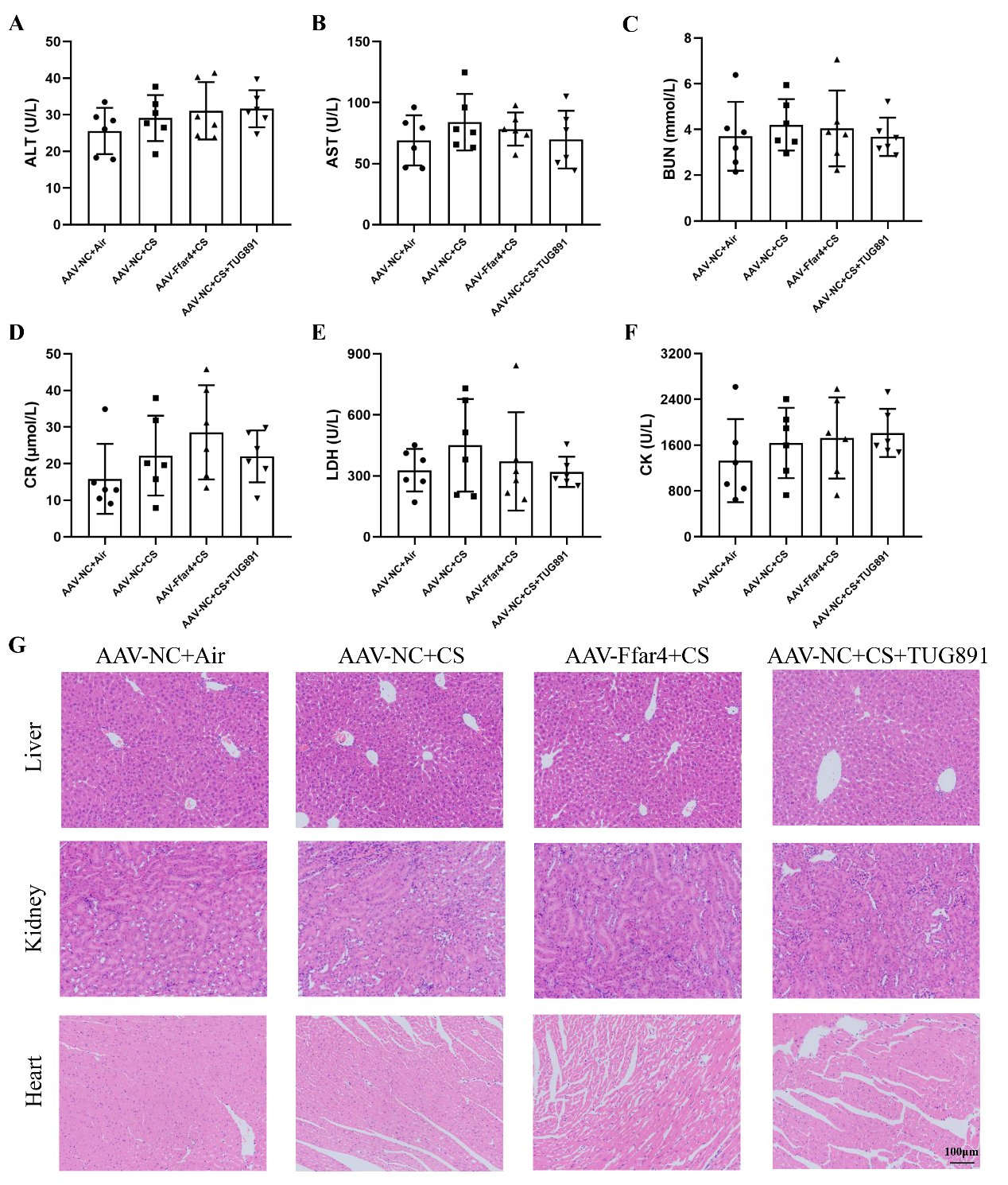


**Figure S2.** The toxic effects of AAV-Ffar4 or TUG891 on the mice.

**(A, B)** Liver function. **(C, D)** Renal function. **(E, F)** Cardiac function. **(G)** The representative images of HE staining in the liver, kidney and heart tissues. Scale bar = 100μm, magnification = x 200. Data were expressed as mean ± SD. P-values were calculated using one-way ANOVA. AAV, adeno-associated virus; NC, negative control; Ffar4, free fatty acid receptor 4; CS, cigarette smoke; ALT, alanine aminotransferase; AST, aspartate aminotransferase; BUN, Blood Urea Nitrogen; CR, creatinine; LDH, lactate dehydrogenase; CK, creatine kinase.

**
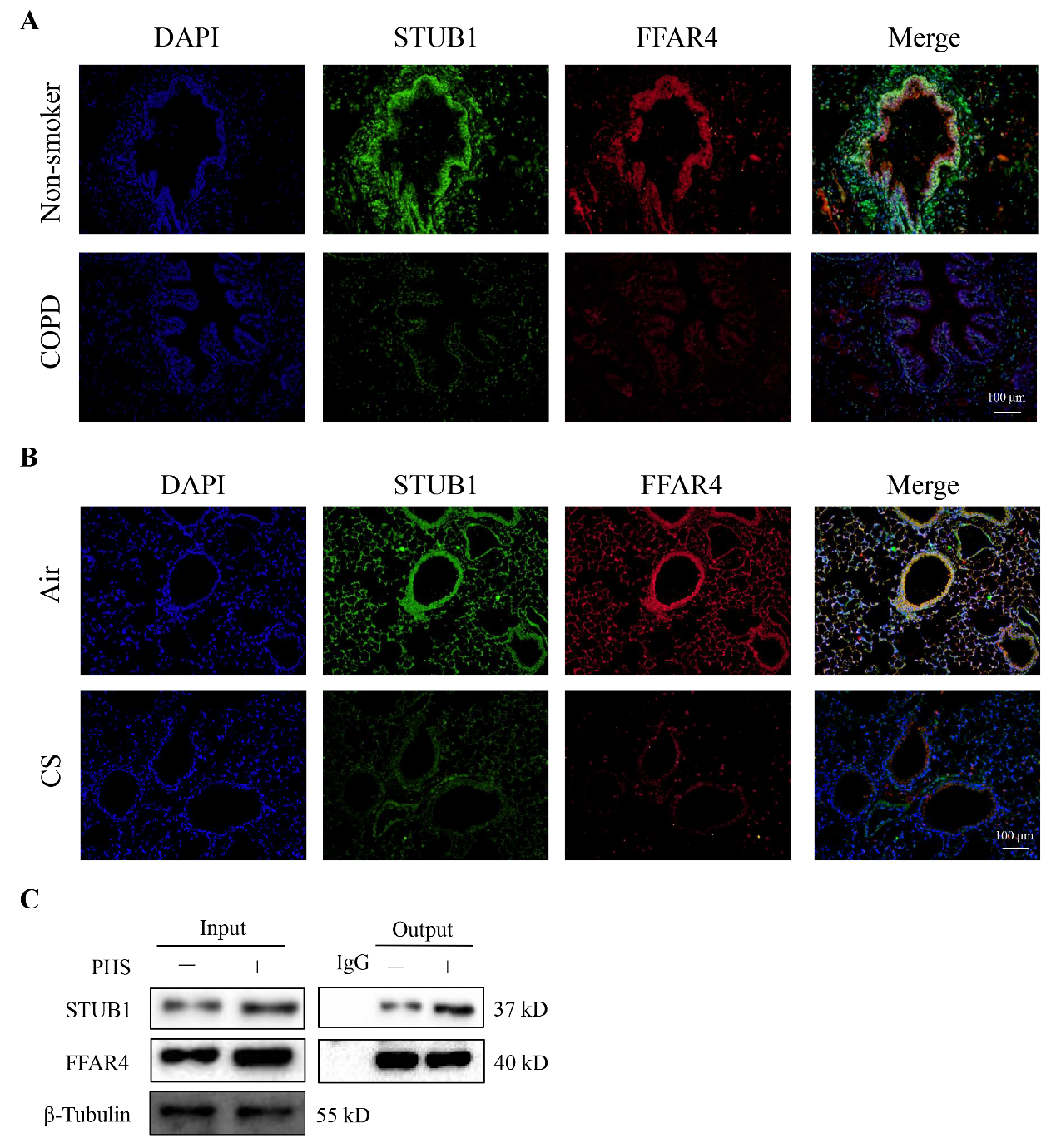
**

**Figure S3.** PHS promoted the co-expression of FFAR4 and STUB1 in the airway epithelial cells.

**(A)** Immunofluorescent co-staining for FFAR4 and P53 in the human lung sections. Scale bar = 100μm, magnification = x 200. **(B)** Immunofluorescent co-staining for FFAR4 and P21 in the human lung sections. Scale bar = 100μm, magnification = x 200. **(C)** Co-immunoprecipitation analysis of FFAR4 and STUB1 in HBE cells with or without PHS treatment. COPD, chronic obstructive pulmonary disease; STUB1, STIP1 homology and U-Box containing protein 1; FFAR4, free fatty acid receptor 4; CS, cigarette smoke; PHS, phytosphingosine; HBE, human bronchial epithelial.


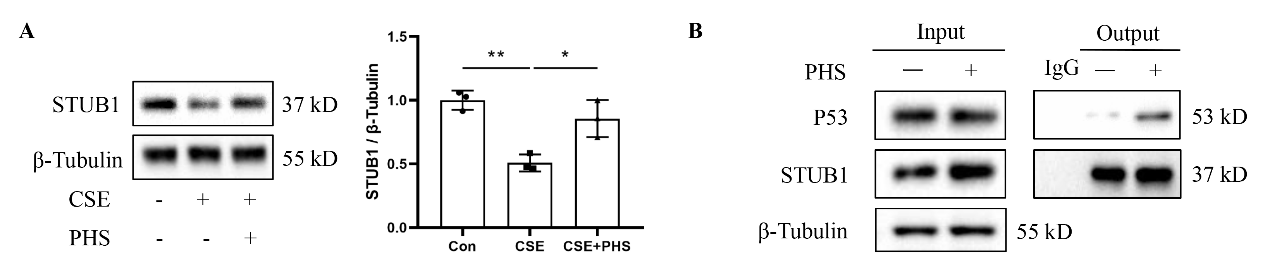


**Figure S4.** PHS promoted the STUB1 expression and combination with P53 in the airway epithelial cells.

**(A)** Western blot analysis of STUB1 in CSE-induced HBE cells treated by PHS. **(B)** Co-immunoprecipitation analysis of STUB1 and P53 in CSE-induced HBE cells with PHS treatment. Data were expressed as mean ± SD. P-values were calculated using one-way ANOVA. **P* < 0.05 and ***P* < 0.01 represented significant differences. STUB1, STIP1 homology and U-Box containing protein 1; PHS, phytosphingosine; CSE, cigarette smoke extract; HBE, human bronchial epithelial.
